# Decline and fall: The causes of group failure in cooperatively breeding meerkats

**DOI:** 10.1002/ece3.7655

**Published:** 2021-10-12

**Authors:** Chris Duncan, Marta B. Manser, Tim Clutton‐Brock

**Affiliations:** ^1^ Department of Zoology University of Cambridge Cambridge UK; ^2^ Kalahari Research Centre, Kuruman River Reserve Van Zylsrus South Africa; ^3^ Animal Behaviour Department of Evolutionary Biology and Environmental Studies University of Zurich Zurich Switzerland; ^4^ Mammal Research Institute University of Pretoria Pretoria South Africa

**Keywords:** cooperative breeding, group failure, group persistence, group size, sociality, tuberculosis

## Abstract

In many social vertebrates, variation in group persistence exerts an important effect on individual fitness and population demography. However, few studies have been able to investigate the failure of groups or the causes of the variation in their longevity. We use data from a long‐term study of cooperatively breeding meerkats, *Suricata suricatta*, to investigate the different causes of group failure and the factors that drive these processes. Many newly formed groups failed within a year of formation, and smaller groups were more likely to fail. Groups that bred successfully and increased their size could persist for several years, even decades. Long‐lived groups principally failed in association with the development of clinical tuberculosis, *Mycobacterium suricattae*, a disease that can spread throughout the group and be fatal for group members. Clinical tuberculosis was more likely to occur in groups that had smaller group sizes and that had experienced immigration.

## Graphical abstract

In social vertebrates, the persistence of groups is important for both individual fitness and population demography. Here, we investigate the causes of group failure in the cooperatively breeding meerkats, showing that larger groups will persist the longest, being buffered against all forms of group failure, and are also less likely to be infected by tuberculosis, a disease often fatal for groups.

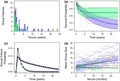

## 1 INTRODUCTION

In social animals, the dynamics of groups can exert considerable influence on the fitness of their members (Riehl, 2011; Woodroffe et al., 2020) and the demography of the population (Angulo et al., 2018). Groups often vary in productivity and persistence and can last for several decades or longer (Alberts & Altmann, 2012; Moss & Lee, 2011). Contrasts in the characteristics, persistence, and productivity of groups can influence the direct fitness of group members (Lardy et al., 2015; Rood, 1990). Moreover, in many social mammals, subordinate females inherit breeding positions in their natal group from their mother. Therefore, successive breeding females are often closely related and matrilineal connections between successive breeding females can persist for several years (Duncan et al., 2018; Pope, 2000). As a result, the persistence of breeding groups (group “longevity”) will also have important effects on the indirect fitness of individuals through the maintenance of their lineage (Akçay & Cleve, 2016).

Currently, relatively few studies of social vertebrates have explored the variation in the persistence of groups, and we know little about the factors that influence their susceptibility to failure. This is especially the case in species with long persisting groups where research has been limited to describing the longevity (Woodroffe et al., 2020) or between‐season survival of groups (Pillay & Rymer, 2017). Groups may fail for multiple different reasons. Sources of mortality including extreme climatic conditions, disease, or predation can either kill all group members (Hanya et al., 2004; McGuire et al., 2002; Sillero‐Zubiri et al., 2015), or reduce groups to a size where they are below the Allee threshold in the presence of strong group‐level Allee effects (Angulo et al., 2018). In addition, group conflict can also cause mortality (Wilson & Wrangham, 2003), particularly in territorial species that display higher rates of interspecific killing (Gómez et al., 2016). The consequences of losing conflicts such as mortality, displacement from valuable territories, and reductions in productivity can all contribute to a group's eventual failure, with smaller groups often being disadvantaged relative to larger groups (Mosser & Packer, 2009). Alternatively, the end of a group as a distinct social unit can occur through fission, with a group splitting into smaller distinct groups or coalitions of dispersers (Alberts & Altmann, 2012; Moss & Lee, 2011). Fission can occur in response to changing ecological conditions (Daniel et al., 2009; Thaker et al., 2010) or due to social perturbations such as the mortality of individuals key to maintaining cohesion between group members (Borg et al., 2015).

Variation in group dynamics has important effects in species where reproductive skew is high, such as the cooperative breeders where either a single female (Clutton‐Brock et al., 2006; Rood, 1990) or a subset of females (Cant et al., 2016) are responsible for most reproduction within the group. Where reproductive skew is particularly high, recruitment at the population level will be more strongly affected by the number of breeding groups than by the number of adult females. Moreover, where the characteristics of groups influence reproduction and survival, they will have considerable impacts on population trends. For example, in cooperatively breeding species, breeding females rely on assistance from other group members to raise their young successfully (Koenig & Dickinson, 2016), and as group size increases, the frequency with which they breed and the survival of their offspring increases (Creel & Creel, 2015; Rood, 1990). This has led to demographic studies of cooperative breeders in recent years incorporating group dynamics and social structure to improve the accuracy with which they capture demographic trends (Zeigler & Walters, 2014). Indeed, where reproductive skew is high, the number of groups rather than the number of adult females becomes the most realistic indicator of the breeding units within a population and can accurately model population demography (Chapron et al., 2016). Therefore, the rates of group failure and formation are likely to determine the recruitment rates and the subsequent trajectory of the population.

In this paper, we explore variation in the persistence of groups in cooperatively breeding meerkats, *Suricata suricatta*, using long‐term data from a population in the Southern Kalahari that have been monitored for 26 years (Clutton‐Brock & Manser, 2016). Meerkats form stable, highly territorial social groups that can persist for many years, consisting of a dominant breeding pair and several subordinates who are usually the dominant's offspring. Dominant females reproductively suppress resident subordinates by killing their offspring and evicting them before they reach full adult weight at 3–4 years (Clutton‐Brock et al., 2010), thus monopolizing their group's reproduction (Clutton‐Brock et al., 2006). Unlike subordinate females, subordinate males voluntarily leave their natal groups at 3–5 years of age to search for breeding opportunities elsewhere, either migrating into other groups or founding new groups with evicted subordinate females (Spong et al., 2008). Dispersing females rarely join established breeding groups and the probability of newly founded groups establishing themselves successfully increases with their coalition size (Maag et al., 2018). Once a group has formed, new females are not able to migrate into the group, and when a dominant female dies, her position will most commonly be inherited by a resident daughter or sibling (Duncan et al., 2018). The tenure of dominant females can last many years, and with multiple successive dominant females holding position, a group can persist for over a decade. Meerkat groups are exposed to multiple risks. In addition to mortality in the forms of predation and starvation (Clutton‐Brock et al., 1999), a fatal species‐specific strain of tuberculosis (TB), *Mycobacterium suricattae*, is endemic within the population (Patterson et al., 2017).

In this study, we aim to characterize the persistence of meerkat groups, identifying the different causes of group failure and quantify their prevalence over time. We also explore the environmental and sociodemographic factors influencing the rate of group failure. We predict that smaller groups will be more likely to fail as they experience increased vulnerability to stochastic mortality (Courchamp et al., 1999), and have reduced helper numbers to mitigate the effects of adverse conditions (Groenewoud & Clutton‐Brock, 2020). However, larger group sizes are commonly associated with increased prevalence of pathogens due to a greater number of social contacts and therefore more opportunities for disease to spread (Altizer et al., 2003). Consequently, we suspect that larger groups of meerkats may be more likely to develop clinical TB which could increase their likelihood of failing. Finally, we test whether groups are more likely to fail in periods of adverse environmental conditions, where the severity of diseases can increase (Summers, 2009) and where groups’ vital rates often decrease (Groenewoud & Clutton‐Brock, 2020).

## 2 METHODS

This study was conducted between October 1993 and May 2019 on groups of meerkats living in the Southern Kalahari. The study site covered 50–60 km^2^ (Cozzi et al., 2018) of semi‐arid desert located on the Kuruman River Reserve and surrounding farms (26°58′S, 21°49′E), Northern Cape, South Africa. Total population size varied from 44 to 365 individuals and was divided between 6 and 24 groups with an average membership of 14 individuals. Study groups were visited 3–5 times a week to collect data, being observable at close proximity with most individuals being approachable to within 1 m. During group visits, detailed data on group membership, health of individuals, reproductive status, and extra‐group movements were collected. All data collected for use in this study were approved by the Animal Ethics Committee of the University of Pretoria, South Africa (EC010‐13), and the Northern Cape Department of Environment and Nature Conservation, South Africa (FAUNA1020/2016).

Over the course of the study, 98 distinct groups were followed, of which 54 were followed until their failure, 14 were still present at the end of the study, and 30 were lost; therefore, their final fates could not be determined. A group was defined as a continuous association of two or more individuals containing members of both sexes. In the rare occasion that all resident males were displaced following the immigration of new males, we considered the group to still exist with the group's identity following the dominant female. A group was considered failed when there were no longer individuals of both sexes present, which could result from either the mortality of all members of one sex within the group or fission between the sexes where the group splits into sex‐segregated dispersing coalitions (often following the death of the dominant female). To account for the role of TB in group failure, we classified group failure into two distinct categories to distinguish between groups that failed without clinical signs of TB and those that failed in association with an ongoing clinical TB infection. Following the development of clinical signs, TB could spread throughout the group, and individuals that developed clinical TB commonly died of natural causes or were euthanised within 6 months (Patterson et al., 2017), resulting in the group declining in size until failure. The exact failure event was observed for most groups in our sample that failed in association with clinical TB. However, due to the mortality of the radio‐collared individual and the emaciated state of the rest of the group, two groups in the final stages of disease could not be followed to failure. There were no sightings of these groups more than 2 weeks after the loss of the radio‐collared individual, and for our analyses, these groups were considered failed on the date the group ceased to be followed.

### 2.1 Assessment of tuberculosis

Tuberculosis (TB) is believed to be endemic in our population and is caused by a species‐specific strain, *Mycobacterium surricattae* (Parsons et al., 2013). The first reports of visual signs associated with TB were recorded in 1998 and were confirmed to be cases of TB by Alexander et al. (2002). Due to the absence of consistent diagnostic sampling for TB across our study, visual indicators of TB were utilized for the identification of clinical infections. The pathology of TB has been extensively investigated, with numerous clincial signs being described, including swollen lymph nodes that present as visible lumps and eventually burst to form lesions (Drewe et al., 2009). Early in the study, we determined that individuals that developed clinical TB died within 6 months of developing signs (Patterson et al., 2017). Since the strain was initially identified as bovine TB and was thought to be a by‐product of cattle ranching, we adopted a practice of euthanising animals whose lymph node swellings burst to reduce the impact of the disease (Patterson et al., 2017). This policy was maintained throughout the study period with 274 individuals euthanized since 1999.

To investigate the role of TB in the failure of groups, we needed to identify the frequency of clinical TB outbreaks in different groups and to separate temporal clusters of clinical TB in group members that were likely to have been the result of the same initial infection event from successive clusters in the same group. Since individuals can remain asymptomatic for many months, it is likely that groups were infected some time before clinical signs of TB were identified in their members (Drewe et al., 2011). To identify the start and end of a temporal cluster of clinical TB, we used individual‐level health records to identify signs indicative of TB and combined them with data on TB‐related euthanasia. To distinguish whether consecutive appearances of clinical TB in a group were likely to have been the result of a single episode of infection or of successive separate infections, we used multistate models to analyze the temporal distribution of successive periods of clinical disease (see Section 1 in Appendix for further details). Periods of clinical infection occurring within a certain timeframe of each other were merged and the model was used to ascertain whether the probability of visible TB signs reoccurring was similar to groups with no history of TB. Based on these analyses, we treated periods of clinical TB occurring more than 13 months apart as distinct periods of clinical infection and periods occurring less than 13 months apart, as part of the same clinical infection. Once the data were analyzed in this way, there were 25 cases in 22 groups where clinical signs of TB did not reoccur within 13 months. To account for the possibility the disappearance of clinical TB was caused by our euthanasia policy, we recalculated our measures of group longevity with groups that experienced the disappearance of clinical TB removed.

### 2.2 Statistical analyses

All analyses were undertaken in the R statistical environment, version 3.5.3 (R Core Team, 2016); survival models were fitted using the packages *survival* (Therneau, 2020) and *flexsurv* (Jackson, 2016) with these models being fitted in a multistate framework using the *mstate* package (de Wreede et al., 2011). Generalized linear mixed‐effect models (GLMMs) and linear mixed‐effect models (LMMs) were fitted using the package *glmmTMB* (Brooks et al., 2017).

To investigate the effect of group number on the reproductive output of the population, our study period was divided into 3‐month periods. The number of pups born within a period was counted and fitted as the response variable in a GLMM with a negative binomial error distribution and zero‐inflation parameter, to account for excess zeros and overdispersion. The quarter of the year the period started in was included as a fixed effect and the year was fitted as a random effect to account for both seasonal and longer‐term environmental variations. Then, the number of groups in the population and the number of adult females (older than a year) were fitted independently as fixed effects.

To investigate whether the early‐life growth trajectories of groups correlated with their cause of failure, we utilized LMMs with a Gaussian error distribution. The monthly mean group size for each group was calculated up until 2 years of age, the development of clinical TB, or their failure, whichever came first. Mean group size was fitted as the response variable. The age of the group in months and whether its failure was associated with a clinical TB infection were included as fixed effects and interacted with each other. Additionally, both a random intercept and slope were included for age and group ID. This model consisted of 471‐month periods from 36 groups. To investigate the early‐life reproductive success of groups, the number of pups produced that emerged from the burrow in the first 2 years of a group's persistence was fitted as the response variable in a GLMM with a negative binomial error distribution. This analysis was restricted to groups that survived long enough to conceive and have pups emerge (3 months). In addition, to account for variation in the amount of time groups had to breed, due to differences in longevity, the number of months a group persisted in the 2 years following formation was included as an offset term in the model. Additionally, to quantify the rearing success of groups, we fitted an additional GLMM with a binomial error distribution and the proportion of pups emerged that survived to nutritional independence (90 days) fitted as the response. To compare groups that failed in association with TB and those that did not, a fixed effect was included in both models with the cause of group failure as a binary factor.

**FIGURE 1:**
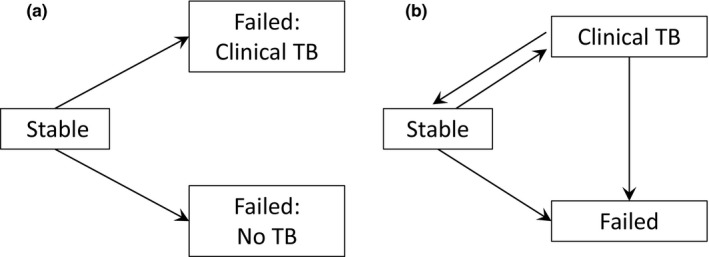
Visual representations of multistate models where boxes represent states a group can occupy and arrows the possible transitions a group can make from one state to another. (a) A competing risk multistate model where groups can transition from being in a stable state to one of either two absorbing states, failure with clinical TB or failure without TB. (b) A multistate illness–death model with recovery, groups can transition from a stable state to having clinical TB and recover again, with failure being the sole absorbing state

For our analyses of group longevity, we restricted our sample to groups whose formation was observed to allow for accurate calculation of age at failure. This resulted in a sample of 62 groups, of which 15 failed without clinical TB, 25 failed in association with clinical TB, and a further 22 that were either still alive or lost before their failure. To characterize the pattern of group failure across time, parametric survival models were fitted. The distributions of best fit were identified by selecting the models with the lowest AIC in combination with visual inspection of the parametric curves against Kaplan–Meier curves (Tables A1, A2 and A1, A2). To investigate variation in patterns of failure relating to the presence or absence of clinical TB, we fitted parametric survival models for each fate; we then fitted these cause‐specific models within a competing risk model, a form of multistate model (Figure 1a). To account for the occurrence of clinical TB being an important precursor step to TB‐associated failure, we also constructed an illness–death multistate model, whereby all groups eventually transition to the same final absorbing state (failure), but groups can transition to an intermediary state representing clinical TB prior to transitioning to failure (Figure 1b). Groups that stopped showing signs of clinical TB transitioned back to a stable state. Groups that were alive at the end of the study or were lost during the study were censored.

To investigate how different factors influence a group's likelihood of failure via different causes, we utilized the illness–death multistate framework (Figure 1b). Semi‐parametric Cox‐proportional hazard models were used, and group lifespans were split into month‐long periods to allow for the fitting of time‐dependent effects that can vary across a group's lifespan. Transition‐specific survival models were used to allow different covariates to be fitted for each transition and to allow the baseline hazard to vary for each transition. As it is possible that the disappearance of clinical TB is influenced by our euthanasia policy, we did not test any fixed effects for this transition leaving it as a null model. Due to the relatively small sample sizes, we conducted univariate models to identify significant variables (Table A3). We then constructed models with the significant variables and the models of best fit were identified using AIC (ΔAIC < 2), and all covariates were tested for violation of the proportional hazard's assumption. Once the best fitting models had been ascertained, we refitted the insignificant variables from the univariate analysis to identify effects that became apparent with other sources of variance controlled for. All continuous variables were mean‐centered and scaled by two standard deviations to allow for comparisons of effect sizes (Gelman, 2008). Excluding periods with missing data our sample consisted of 2148 months with groups in the stable state from which 41 transitions to a clinical TB state and 14 to a failed state were observed, and 466 months with groups in a clinical TB state from which 20 transitions to failure and 19 to a stable state were observed.

To test for the influence of climate on all transitions, we used standardized precipitation indices (SPI) calculated monthly with remotely sensed rainfall estimates from the NOAA Global Precipitation Climatology Project (Adler et al., 2018). SPI is a drought index which quantifies how precipitation deviates from the monthly mean based on historical data (Mckee et al., 1993). The period over which SPI can be calculated can be adjusted to test short‐ and long‐term variations in precipitation. We tested a range of SPI measures from 1 to 12 months to see whether climate had a direct effect on these processes and whether short‐ or long‐term precipitation was more important. Further details on the calculation of this measure for our system can be found in Groenewoud and Clutton‐Brock (2020). We also fitted season as a binary effect, with the months from October to March, where rainfall and reproduction are the highest, classified as the “high season” and April to September as the “low season.”

The effect of sociodemographic factors on the different transitions was also investigated. For the transition of groups in a stable state to failure and clinical TB, we included mean group size and population density as time‐varying fixed effects. Density was calculated as the monthly population size divided by the total study area derived from 95% kernel density estimations using all burrows used by the population within the month (Cozzi et al., 2018; Paniw et al., 2019), using the R package *adehabitat* (Calenge, 2006). Previous research suggests that TB is spread between meerkat groups via dispersal events (Drewe, 2010). We therefore included variables representing whether new individuals had immigrated into the group and the number of out‐of‐group excursions group members had made. The period over which immigration and excursions were calculated was back cast in time to account for the fact a group's infection event likely occurred prior to visual signs of TB becoming apparent. To avoid setting an arbitrary period to measure immigration and excursions, we refitted our models with periods ranging from 1 to 16 months prior and selected the time period with the lowest AIC value (Table A6). Taking this into account, we fitted our final models with a binary term for whether immigration had occurred within the previous 7 months and a term for the number of excursions occurred within the previous 6 months. For the transition from TB to failure, group size was fixed at the time clincial TB occurred, to avoid modeling the expected terminal decline as group members die from disease. Additionally, to test whether recruitment of new group members via reproduction allowed groups to postpone their failure, we included their reproductive rate (number of pups survived to 90 days/number of days in state) during the period of clinical TB as a time fixed variable.

To increase the power of the analysis investigating the progression of groups with clinical TB to failure, we constructed a separate Cox‐proportional hazard model. The time from the development of clinical TB to failure was fitted as the response variable. This allowed us to increase our sample size by including groups of unknown formation date for which we had observed the development of clinical TB, as progression was relative to the onset of clinical TB not group formation. The model was fitted with the same covariates that were tested on the transition from TB to failure in the multistate model. The sample for this analysis consisted of 580 months with groups in a clinical TB state from which 25 groups failed (Table A7).

## 3 RESULTS

### 3.1 Group number and recruitment

**FIGURE 2:**
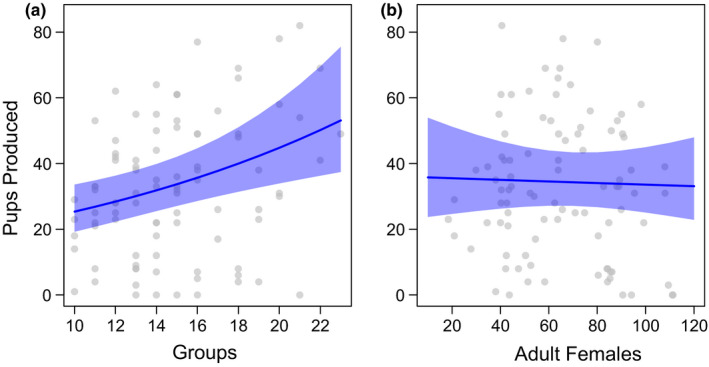
The relationship between the number of pups produced that emerged in our population during 3‐month periods and the number of (a) groups and (b) adult females in the population. Raw data plotted as gray points and the model predictions plotted as solid blue lines with accompanying confidence intervals shaded in blue. All predictions derived at the population level with the time of year set to the third quarter (July–September), from GLMMs with a negative binomial error distribution and a zero‐inflation term. The dataset included 92 periods from October 1996 to October 2019

As a single female (virtually) monopolizes reproduction in each group, the total productivity of the population was more closely related to changes in the number of groups in the population than to the numbers of adult females. With seasonal and between‐year variation in reproduction accounted for (Table A4), the number of pups produced increased as the number of groups in the population increased (Figure 2a, GLMM: Est ± *SE* = 0.057 ± 0.018, *p* = .001), while the number of adult females present in the population had no effect (Figure 2b, GLMM: Est ± *SE* = 0.001 ± 0.003, *p* = .807).

### 3.2 Group longevity and failure

**FIGURE 3:**
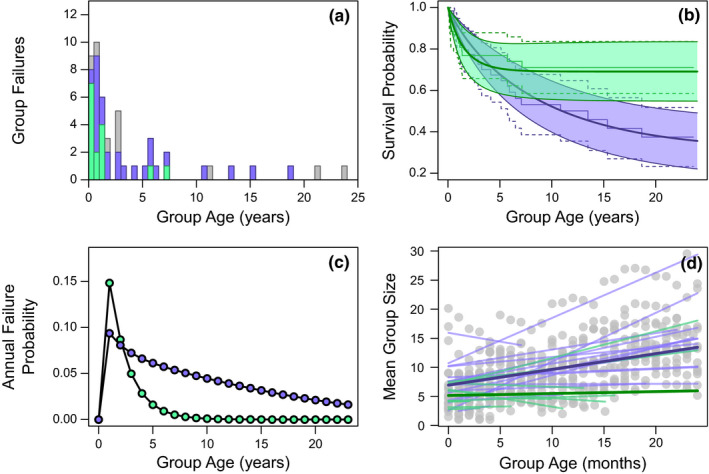
The variation in group characteristics across existence in relation to the group's cause of failure, with failures relating to TB (purple) and failures of groups with no TB (green) represented. (a) Stacked frequency plot of age at failure with groups still alive included with their age at study end (gray). (b) Survival probability across a group's existence with 95% confidence intervals (shaded area) predicted from parametric competing fate survival models overlaid on raw Kaplan–Meier plots with 95% confidence intervals (dashed lines). (c) The annual probability of a group failing across their existance derived from parametric survival models. (d) The mean monthly group size across the first 2 years of persistence with raw data (gray points), population‐level predictions (thick lines), and group‐level predictions (lighter thin lines) plotted

Three groups have been observed to survive for longer than 20 years, of which two were still active at the end of this study. However, the distribution of group longevities for groups followed from formation to failure was left‐skewed with a median longevity of 1.17 years (*n* = 40, mean = 3.32 years, range = 0.08–18.66 years; Figure 3a). Excluding groups that had experienced a recovery from clinical TB, the median longevity was 0.97 years and groups could still persist for over a decade with a maximum observed longevity of 13.5 years (*n* = 30, mean = 2.18 years, range = 0.08–13.5). Of the 54 groups whose failure was observed, 37% failed with no signs of clinical TB; of these groups, 13 failed because of fission between the sexes and 7 following a mortality event that killed all the members of one sex. The risk of failure not associated with TB was highest in the first year after a group formed, with the rate of failure declining to negligible levels within 5 years (Figure 3b,c). These groups tended to show no growth following formation, remaining small in size, possibly explaining their subsequent failure and short persistence (Figure 3d, Table A5). This was in contrast with the pronounced growth in size, groups that failed in association with TB displayed prior to the development of clinical disease (Figure 3d, Table A5). The absence in growth appears to be due to the reduced rate of reproduction these groups experienced (GLMM: *n* = 33, Est ± *SE* = −3.814 ± 1.482, *p* = .01, as they were not significantly smaller at formation (GLMM: *n* = 31, Est ± *SE* = −0.207 ± 0.186, *p* = .26) and their offspring did not experience significantly reduced rates of survival to independence (LM: *n* = 28, Est ± *SE* = −0.752 ± 0.848, *p* = .40).

**FIGURE 4:**
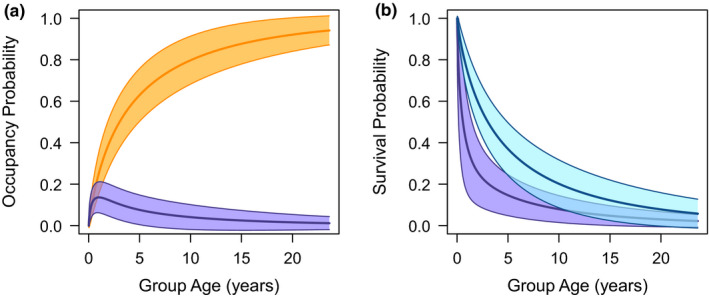
Model predictions from the parametric multistate illness–death model. (a) Predicted occupancy probabilities for a group having clinical TB (purple) and a group having failed (orange) across their lifespan. (b) The survival probability across a group's lifespan depending on whether they have clinical TB (purple) or not (blue)

The remaining 63% of groups failed in association with clinical TB and the risk of failure with clinical TB peaked after formation and gradually declined over time (Figure 3b). For groups that survived beyond a year, TB became the most probable cause of failure, and of the seven groups that failed with longevities greater than 10 years, all failed with clinical TB (Figure 3a,c). When modeled within an illness–death framework, the probability of a group developing clinical TB was approximately stable across a group's lifespan following a peak early in life (Figure 4a). Once groups developed clinical TB, their survival probability decreased considerably compared with that of groups without visual signs (Figure 4b). For groups that failed with clinical TB, their mean survival time after the development of visible signs was 10 months.

### 3.3 Causes of failure

For groups without clinical TB, smaller groups and those experiencing a higher population density were more likely to fail. Group size exerted a strong negative effect on the probability of group failure, whereas population density had a positive effect (Table 1). Smaller groups were also significantly more likely to develop clinical TB, along with groups that had increased exposure to out‐of‐group individuals (Table 1). Immigration events within the previous 7 months had a significant positive effect on the probability of developing clinical TB. In addition, the number of excursions individuals undertook outside the group within the previous 6 months also had a positive effect on the probability of developing TB, although this was not significant (Table 1). Most groups that developed clinical TB failed, however, groups that were larger at the onset of clinical TB and had a higher recruitment rate during the period of clinical infection survived for longer. The negative effects of group size and recruitment rate on survival with clinical TB for groups of known formation date, were not significant in both univariate models (Table A3) and when fitted together (Table 1). However, when this transition was modelled with a larger dataset including all observed bouts of clinical TB, including groups of unknown formation date, both the negative effect of group size and recruitment rate were statistically significant (Tables 1 and A7). There was evidence that groups that developed clinical TB were less likely to fail when precipitation was above average in the preceding 6 or 12 months (SPI6 & SPI12, Table A3). Although this effect remained statistically significant only for precipitation in the previous 12 months when the larger dataset including all TB bouts was modelled, yet this effect was relatively small compared to other covariates (Tables 1 and A7). Beyond this, environmental factors had little impact on group failure and neither season nor the standardized precipitation index had a significant effect on any of the other transitions within our models (Table A3).

**TABLE 1:**
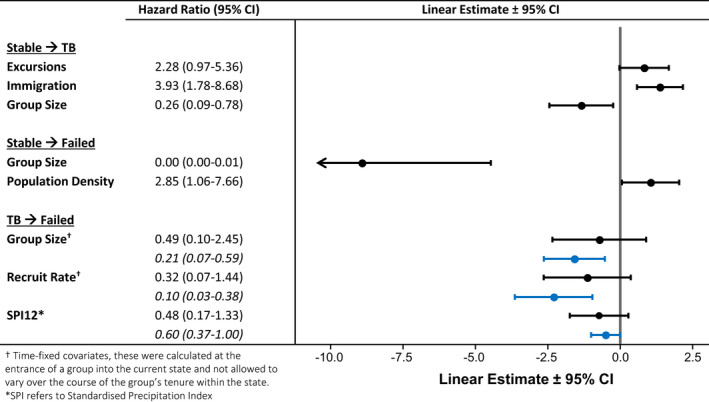
Model outputs and forest plot for semi‐parametric multistate illness–death model of meerkat group survival (black points and lines). In addition the model outputs for the remodeled transition from clinical TB to failure on a larger dataset (Table A7), including groups of unknown formation are also reported (italics) and included in the forest plot (blue points and lines)

## 4 DISCUSSION

Meerkat groups are exposed to a high risk of failure in the first few years of their life with many groups failing. However, groups that survive this period can experience longevities of over a decade. Long‐lived groups primarily failed in association with a clinical TB infection that increased their likelihood of failure. The persistence of groups is important for maintaining meerkat populations, as due to the high levels of reproductive skew, the number of groups rather than the number of breeding age females better predicts the reproductive output of the population. The survival and persistence of groups are strongly influenced by sociodemographic factors, particularly their group size which positively influences group survival. Larger groups are less likely to fail, develop clinical TB, and will survive with clinical TB for longer. The influence of climate on the persistence of groups was also investigated, yet contrary to our expectations, this did not appear to influence the occurence of clinical TB or the failure of groups without clinical signs of TB. There was evidence of groups with TB being less likely to fail when precipitation had been above average in the previous year, however this effect was relatively small.

The positive relationship between the persistence of groups and their size supported our prediction and has been previously described in several other vertebrates, including the African ice rat, *Otomys sloggetti robertsi* (Pillay & Rymer, 2017), Arabian babblers, *Turdoides squamiceps* (Keynan & Ridley, 2016), and cichlids, *Neolamprologus pulcher* (Jungwirth & Taborsky, 2015). Smaller groups are often more vulnerable to mortality events (except see McGuire et al., 2002), experiencing mortality at a higher rate and with individuals key to maintaining group cohesion exposed to a greater mortality risk due to a reduction in the predator dilution effect (Courchamp et al., 1999). Smaller groups of meerkats have also been shown to experience higher per capita mortality (Bateman et al., 2012), as well as reduced dominant reproductive success (Hodge et al., 2008). The effect of these constraints was visible in our data as groups that were smaller at formation and reproduced less successfully experienced little growth, therefore remaining small in size and vulnerable to failure.

Group failure also occurred due to fission between the sexes. The failure of smaller groups in this way may be a result of the reduction in survival and reproduction that smaller groups experience, making residency costly with few fitness benefits. Individuals may therefore benefit by abandoning their group, triggering its failure, to undergo a secondary dispersal to found or migrate into a larger group with better fitness prospects. Possibly, even to return to their natal group, obtaining inclusive fitness by providing care for relatives’ offspring. The same processes could also explain why groups were more likely to fail during periods of high population density. Increases in population density can lead to the saturation and overlap of territories (López‐Sepulcre & Kokko, 2005; Ridley et al., 2004), resulting in increases in the rate and severity of intergroup conflict (Wilson et al., 2014). The consequences of increasing conflict including decreases in territory quality, productivity, and survival, may generate situations where it benefits individuals to abandon their group. These effects are also believed to operate on dispersing meerkats, explaining why as population density increases, emigration and settlement rates decline while the rates of dispersers returning to their previous groups increase. Although it seems at the highest population densities, these effects begin to reverse (Maag et al., 2018). We suspect that the negative effect of population density will be biased toward relatively smaller groups as they are more likely to lose intergroup interactions (Dyble et al., 2019) and therefore would disproportionately suffer the consequences of increased rates of competition. However, our sample sizes were too small to test for the existence of this effect.

The groups that avoided failure early in their lifespan displayed increased rates of successful reproduction and were able to grow their group size considerably during their first 2 years, going on to persist for many years. Of the groups that survived beyond a year, the majority failed following the development of clinical TB infections that greatly increased a group's risk of failure. By driving the failure of long persisting groups that play an important role in recruitment and emigration within the population, TB plays an important demographic role for meerkats. Much of the demographic variation in meerkats will be explained by the balance between groups failing from TB and the formation rate of new groups to replace lost breeding units.

Corroborating previous research on meerkats, we show that the levels of contact with outgroup individuals predict the risk of groups developing observable signs of a TB infection (Drewe, 2010). In addition to confirming the positive effect male immigration events have on TB developing (Patterson et al., 2017), we also found some evidence that the number of individual excursions away from the group, possibly interacting with infected individuals or groups, may also increase a group's likelihood of clinical TB. Mobile individuals, such as dispersers and prospectors, can be important drivers of pathogen transmission (see badgers *Meles meles* Vicente et al., 2007; lizards, *Tiliqua adelaidensis* Fenner et al., 2011), as they create infection pathways between groups and are often exposed to a diversity of individuals (Nunn et al., 2008). However, this is not universal. In lions, highly mobile “nomad” individuals have little impact on disease transmission, with transmission instead occurring between neighboring groups (Craft et al., 2011).

With the levels of extra‐group movement controlled for, we also found that smaller groups were more likely to develop clinical TB. This was in contrast to our expectations and the general perception that a cost of increasing group size and sociality is the increased exposure to pathogens (Altizer et al., 2003). While we cannot be sure the effect of group size on the development of clinical disease translates to the risk of infection, negative effects of group size on disease prevalence have been observed in other species (Keiser et al., 2018; Woodroffe et al., 2009). Moreover, it is hypothesized that the increasingly modular organization of larger groups could reduce disease prevalence (Nunn et al., 2015), with social clustering that could limit disease transmission being reported in meerkats (Drewe et al., 2011). However, these mechanisms influence the prevalence rather than the presence of disease, largely explaining the patterns of spread within and not between groups. Therefore, while this could explain larger groups of meerkats persisting longer with clinical TB, it does not explain why larger groups are less likely initially to develop clinical TB. We suggest that the increased probability of clinical TB occurring in smaller groups could be a function of how they interact with other groups and coalitions of dispersers. For example, in badgers it has been suggested that increased TB prevalence in smaller groups could result from smaller groups having higher contact with neighboring group members (Woodroffe et al., 2009). Variation in the nature of these interactions could also influence the likelihood of transmission. If smaller groups are more likely to experience intergroup interactions that escalate to physical aggression, this could increase the risk of transmission, as aggressive behaviors such as biting are possible transmission pathways (Drewe, 2010).

Though climatic factors can play an important role in disease dynamics (Summers, 2009), influencing the prevalence and lethality of infections (Munson et al., 2008; Randall & Van Woesik, 2015), we found no evidence of direct relationships between season and precipitation on the emergence of clinical TB in groups. This differed from our prediction and a previously reported weak effect indicating TB incidence was higher in drier periods (Patterson et al., 2017). However, as we were unable to measure the exact time of a group's infection with TB, only the development of clinical signs, this could have prevented us from detecting the effects of climate on TB spread. We suspect that climatic variation will be involved in indirectly driving the process of TB infection. For both group size and the rates of extra‐group movement, predictors of the development of clinical TB have been shown to be influenced by seasonal and climatic variation (Bateman et al., 2013; Mares et al., 2014). Considering the importance TB has for the persistence of meerkat groups, understanding how climate operates through these parameters to influence disease prevalence will be essential for predicting the future population trends in response to changing climatic conditions.

The practice of targeted euthanasia for individuals with clinical TB is likely to impact on the persistence of groups in our population. For example, the premature removal of infectious individuals could prevent the spread of disease to other groups and possibly stem the further spread and development of TB within groups. While it is not known whether euthanasia prevented further development of TB within some groups, if it is the case, then the median and maximum observed longevity of groups would be artificially increased in our population. In contrast, euthanasia could also accelerate a group's progression from developing clinical signs to failure. However, as individuals were euthanised in the later stages of what appears to be a terminal infection, we suspect euthanasia rarely curtails a group's persistence by more than a few months. More broadly, while euthanasia may influence the longevity of groups, we believe that our results describing the general patterns of group persistence and how they are affected by group characteristics will be largely unchanged and will still be relevant beyond our population.

In conclusion, we characterize the longevities of meerkat groups and identify the causes of their failure, revealing the influence of TB on meerkat demography by driving the failure of the longest‐lived groups. Additionally, we show the importance of large group sizes for reducing a group's risk of failure, with groups that grow quickly after formation and sustain a large group size persisting the longest. Studies, such as ours, that investigate the processes of group survival and their eventual failure provide valuable information for understanding and modeling the demography of social species. As sample sizes continue to grow, we believe that extending this work to investigate how the composition of groups and the characteristics of their members influence group persistence will be valuable.

## CONFLICT OF INTEREST

No conflicts of interest to declare.

## AUTHOR CONTRIBUTIONS

**Chris Duncan:** Conceptualization (equal); Data curation (equal); Formal analysis (lead); Methodology (lead); Writing‐original draft (lead); Writing‐review & editing (equal). **Marta B. Manser:** Data curation (equal); Funding acquisition (equal); Project administration (equal); Resources (equal); Writing‐review & editing (equal). **Tim Clutton‐Brock:** Conceptualization (equal); Data curation (equal); Funding acquisition (equal); Project administration (equal); Resources (equal); Supervision (equal); Writing‐review & editing (equal).

## Figures and Tables

**FIGURE A1:**
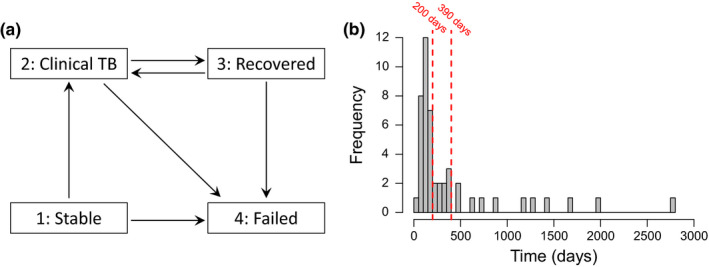
Multistate model design, an expanded illness‐death model with an additional state for groups that stopped displaying clinical signs of TB (a). The distribution of time to recurrence of clinical TB plotted as a histogram (b)

**FIGURE A2:**
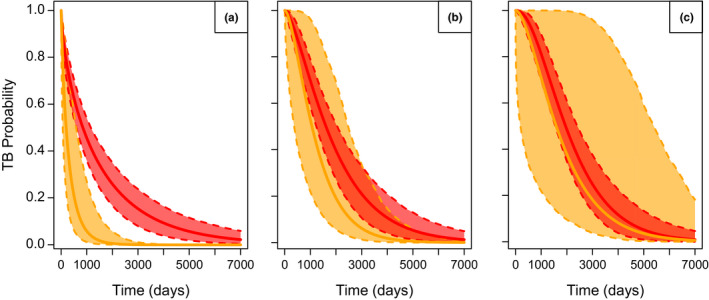
Parametric survival curves for the probability a group enters a clinical TB state, depending on whether the group had no prior history of clinical TB (red) or had returned to a recovered state after experiencing a period of clinical TB (orange). All models are accelerated time failure models fitted with a gamma distribution; goodness of fit was checked visually against Kaplan–Meier plots in all cases. The curve predicted by the model (solid line) along with 95% confidence intervals (translucent polygon with dashed borders) were plotted for all models. Plot (a) represents the raw data where groups with clinical TB infections that do not display visual signs of TB for a month are transitioned to a recovered state, for plot (b) groups that have clinical TB reoccur within 200 days are kept in the TB state between the clinical periods and for plot (c) groups that have clinical TB reoccur within 390 days are kept in the TB state between the clinical TB periods. As for plots (b) and (c), recovered groups had to have survived for a certain period without visual signs of TB, by definition, they were only compared against naïve groups that had survived at least that long without an infection

## Data Availability

Data used for analyses are available on Dryad Digital Repository at https://doi.org/10.5061/dryad.66t1g1k1r.

## 1 correcting fragmentation of periods of continuous tb infection

To merge periods of clinical TB that were likely part of the same underlying group infection, we utilized a multistate model that transitioned groups that no longer displayed signs of clinical TB as a “recovered” state (Figure A1). We then created datasets where distinct periods of clinical TB occurring within a certain time period of each other were merged and treated as a single continuous period of clinical TB. Datasets were created with cutoffs for merging periods of clinical TB ranging from 0 to 24 months at 1‐month increments (30 days). Beyond 2 years, our data was too sparse for modeling the recurrence of clinical TB. These datasets were then fitted to parametric survival models, and the survival curves for the transition to TB from a “recovered” state and a “stable” state (groups with no prior clinical TB) were compared (Figure A2).

Fitting models with the raw data and all periods of clinical TB treated as separate, we found that the probability of a recovered group returning to a TB state was highly likely (Figure A2a). This is to be expected if the disease was present in a latent state at the group. When TB periods <13 months apart were merged and the models were rerun, the groups that recovered from TB had a similar TB probability across time to stable groups (Figure A2c). While we cannot be sure that the TB infection disappeared from the group without molecular diagnostic monitoring, this suggested that clinical TB infections more than 13 months apart were likely two separate infections. Models ran with datasets where the merging cutoff was >13 months gave similar results showing no increased likelihood of clinical TB reoccurring compared with groups with no prior history. For datasets where the merging cutoff was <13 months, the “recovered” groups were more likely to redevelop clinical TB than groups with no prior history of TB (Figure A2b), especially at lower cutoffs (<6 months).

To test the robustness of our multistate illness‐death model (Table 1) to the assumption that periods of clinical TB greater than 13 months apart were distinct, we reran our models on additional datasets. We fitted datasets with TB periods merged at across greater time periods (2 – 5 years in yearly increments), which results in more conservative estimates of distinct infections. Furthermore, we ran a model where only the first occurrence of clinical TB was modelled, thereby making no assumptions about subsequent periods of clinical TB. These models largely yielded results in concordance with our model presented in this paper (Tables 1 and A8). The direction and strength of all the effects were conserved with periods of distinct clinical TB occurring within up to 5 years of each other being treated as the same clinical infection, with only negligible changes in statistical significance, which likely result from decreasing sample size (Table A8). Only in the most conservative model, where groups were considered to have clinical TB from first occurrance irrespective of how long they returned to non‐clinical state for, was a non‐negligible change in effect sizes observed (Table A8). However, the directionality of the effects in this conservative model was still consistent with our previous models, and we suspect this treatment is overly conservative as it treats two sets of distinct clinical TB separated by 5.4 years and 7.4 years in which no clinical TB was observed as the same underlying infection. Therefore, we believe the results of our model to be robust and not a function of the way we have treated these data.

**TABLE A1:** Model comparisons for competing risk survival analyses characterizing the failure of groups accounting for two distinct fates (TB vs. No TB) in addition to censoring. The models with the lowest ΔAIC value were considered the model of best fit and are highlighted in bold

	No TB			TB		
	AIC	LogLik	ΔAIC	AIC	LogLik	ΔAIC
Exponential	114.13	−56.06	14.94	**163.34**	**−80.67**	**0**
Weibull	102.47	−49.23	3.28	164.73	−80.36	1.39
Gamma	103.46	−49.73	4.28	164.97	−80.49	1.63
Log normal	99.25	−47.62	0.06	163.36	−79.68	0.02
Gompertz	**99.19**	**−47.6**	**0**	164.21	−80.1	0.87
Log logistic	101.25	−48.63	2.06	163.86	−79.93	0.52

**TABLE A2:** Model comparisons for parametric time homogeneous survival models for each transition in the multistate illness‐death model

	Stable → TB			Stable → Failure		
	AIC	LogLik	ΔAIC	AIC	LogLik	ΔAIC
Exponential	810.89	−404.45	4.82	281.13	−141.57	9.85
Weibull	806.53	−401.3	0.46	274.8	−135.4	3.52
Gamma	**806.07**	**−401.04**	**0**	275.91	−135.95	4.63
Log Normal	817.59	−406.8	11.52	**271.28**	**−133.64**	**0**
Gompertz	NA	NA	NA	NA	NA	NA
Log Logistic	817.95	−407	11.88	273.34	−134.67	2.06

	TB → Stable			TB → Failure		
	AIC	LogLik	ΔAIC	AIC	LogLik	ΔAIC
Exponential	**294.51**	**−146.25**	**0**	373.16	−185.58	1.72
Weibull	295.93	−145.96	1.42	**371.44**	**−183.72**	**0**
Gamma	295.72	−145.86	1.21	372.63	−184.31	1.19
Log Normal	300.87	−148.44	6.36	371.55	−183.78	0.11
Gompertz	NA	NA	NA	NA	NA	NA
Log Logistic	304.2	−150.1	9.69	372.91	−184.46	1.47

Note

For some transitions, models fitted with a Gompertz distribution could not be fitted due to convergence issues; these are represented by NAs. The models with the lowest ΔAIC value were considered the model of best fit and are highlighted in bold.

**TABLE A3:** Univariate model outputs for semi‐parametric Cox proportional hazard models fitted within the multi‐state illness‐death model

	Alive → Dead		Alive → TB		TB → Dead	
	Est ± *SE*	*p*	Est ± *SE*	*p*	Est ± *SE*	*p*
Group size	−7.215 ± 1.889	<.001	−1.109 ± 0.484	.022	−1.32 ± 0.768	.086
Pop density	0.175 ± 0.515	.734^*^	−0.558 ± 0.080	.136	−0.106 ± 0.128	.409
Immigrants	NA	NA	1.489 ± 0.395	<.001	NA	NA
Excursions	NA	NA	0.195 ± 0.350	.578^*^	NA	NA
Recruit rate	NA	NA	NA	NA	−1690 ± 0.873	.053
SPI1^a^	0.417 ± 0.297	.166	−0.002 ± 0.198	.992	−0.261 ± 0.309	.397
SPI3^a^	0.056 ± 0.287	.845	−0.190 ± 0.168	.26	−0.533 ± 0.325	.101
SPI6^a^	−0.081 ± 0.278	.772	−0.228 ± 0.173	.186	−1.214 ± 0.518	.019^b^
SPI12^a^	−0.218 ± 0.347	.53	−0.074 ± 0.189	.697	−1.029 ± 0.512	.044
Season	0.350 ± 0.551	.525	−0.313 ± 0.326	.338	−0.397 ± 0.547	.468

^a^SPI refers to the Standardised Precipitation Index.

^b^When modeled with a larger dataset including all transitions from TB to death, the significance of this effect no longer held (Haz [CI 95%] = 0.78 [0.50–0.23], Est ± *SE* = −0.24 ± 0.23, *p* = .29).

*These terms were not significant when modeled with a univariate approach; however, when the terms were refitted with the significant terms, it became apparent that these terms had an effect once the effect of group size had been accounted for. Models fitted better with these terms included according to AIC values.

**TABLE A4:** Model outputs for GLMMs investigating the factors influencing the number of pups produced in the population during 3 month periods over the course of the study

	Estimate ± *SE*	*z*‐Value	*p*
Group model			
Group number	0.057 ± 0.018	3.243	.001
Quarter (Set to 1st)			
2nd Quarter	−1.115 ± 0.163	6.861	<.001
3rd Quarter	−0.304 ± 0.154	1.975	.048
4th Quarter	−0.154 ± 0.150	1.029	.303
Zero‐inflation term	−2.870 ± 0.474	6.160	<.001
Females model			
Adult female count	−0.000 ± 0.003	0.244	.807
Quarter (Set to 1st)			
2nd Quarter	−1.137 ± 0.173	6.562	<.001
3rd Quarter	−0.295 ± 0.166	1.774	.076
4th Quarter	−0.170 ± 0.160	1.059	.290
Zero‐inflation term	−2.888 ± 0.474	6.090	<.001

**TABLE A5:** LMM model outputs for the effect of covariates on group size variation in the first 2 years of a group's longevity

	Estimate ± *SE*	*z*‐Value	*p*
Age^a^	0.238 ± 1.102	0.296	.768
Failure cause	4.234 ± 1.333	3.176	.002
Age:Failure cause	1.691 ± 0.945	1.790	**.** **0**74

^a^ The Age of a group is the time in months since the formation of the group.

## 2

**TABLE A6:** Model comparison table for the effect of excursions and immigration on the likelihood of clinical TB developing calculated over different timeframes

Months	Excursions		Immigration	
	AIC	ΔAIC	AIC	ΔAIC
1	201.31	2.90	207.41	6.16
2	201.69	3.29	206.88	5.63
3	201.51	3.10	210.08	8.83
4	200.23	1.83	208.13	6.87
5	199.12	0.71	206.21	4.96
6	**198.40**	**0.00**	207.99	6.74
7	198.99	0.59	**201.25**	**0.00**
8	199.96	1.56	203.59	2.33
9	200.22	1.81	204.11	2.86
10	200.24	1.83	206.60	5.35
11	200.48	2.08	205.33	4.07
12	200.12	1.72	205.63	4.38
13	200.33	1.93	206.70	5.45
14	200.36	1.96	206.92	5.66
15	200.09	1.69	207.97	6.71
16	199.95	1.55	209.34	8.09

Note

The time period producing the model of best fit are highlighted in bold.

## 3 OUTPUTS FOR MODEL INVESTIGATING THE TIME FROM ONSET OF CLINICAL TB TO FAILURE INCLUDING ALL GROUPS OBSERVED TO DEVELOP TB

To further investigate the transition of groups with clinical TB to failure, we fitted a Cox‐proportional hazard model with entry into the study as the time clinical TB developed and the event of interest being failure. This allowed us to increase our sample size compared with the multistate model, for we could include groups of unknown formation time as we knew the time of clinical TB onset relative to failure. This analysis included a sample of 580‐month observations of groups with clinical TB during which 25 groups observed to fail. This analysis revealed that the negative effects of group size, recruitment rate and SPI12 on the likelihood of a group with TB failing was significant (Tables 1 and A7). While SPI6 had a significant negative effect on this transition in our model restricted to groups of known formation (Table A3), this effect was not replicated in this analysis with group size and recruitment rate accounted for (Haz [CI 95%] = 0.78 [0.50 – 0.23], Est ± SE = −0.24 ± 0.23, *p* = .29).

**TABLE A7:** Outputs for Cox proportional hazard model fitted on the time to failure following the development of clinical TB

	Hazard Ratio (95% CI)	Linear estimate ± *SE*	*p*
TB → Failed			
Group size^a^	0.20 (0.07–0.59)	−1.584 ± 0.538	.003
Recruit rate^a^	0.10 (0.03–0.38)	−2.299 ± 0.684	<.001
SPI12	0.60 (0.37–1.00)	−0.506 ± 0.257	.048

^a^Are time‐fixed covariates, these were calculated at the development of clinical TB.

**TABLE A8:** Multistate nonparametric survival model outputs for the transition from stable state to clinical TB, modeled with distinct periods of clinical TB that occurred within a set number of months of a previous clinical TB period merged and treated as a continuous clinical TB infection

Model	Events	EST	*SE*	Haz [95% CI]	*p*
Immigration					
13 months	41	1.369	0.404	3.93 [1.78–8.68]	<.001
24 months	38	1.405	0.425	4.07 [1.77–9.38]	<.001
36 months	38	1.405	0.425	4.07 [1.77–9.38]	<.001
48 months	35	1.485	0.44	4.41 [1.86–10.45]	<.001
60 months	34	1.441	0.445	4.22 [1.76–10.12]	.001
Full Merge	32	1.626	0.489	5.08 [1.95–13.28]	<.001
Group Size					
13 months	41	−1.346	0.563	0.26 [0.08–0.78]	.018
24 months	38	−1.251	0.584	0.28 [0.09–0.90]	.032
36 months	38	−1.251	0.584	0.28 [0.09–0.90]	.032
48 months	35	−1.163	0.616	0.31 [0.09–1.04]	.059
60 months	34	−1.107	0.627	0.33 [0.10–1.13]	.077
Full Merge	32	−0.904	0.637	0.41 [0.12–1.41]	.156
Excursions					
13 months	41	0.824	0.436	2.28 [0.97–5.36]	.059
24 months	38	0.941	0.47	2.56 [1.02–6.44]	.046
36 months	38	0.941	0.47	2.56 [1.02–6.44]	.046
48 months	35	0.89	0.526	2.41 [0.86–6.74]	.095
60 months	34	0.901	0.571	2.46 [0.80–7.55]	.11
Full Merge	32	0.512	0.541	1.67 [0.58–4.83]	.344

Note

Different timeframes for merging TB periods were modeled, ranging from 13 to 60 months in yearly intervals, with the results present here. 13 months is the timeframe over which clinical TB periods were merged in the main paper. Full merge refers to a model where only the first occurrence of clinical TB was modeled, and all subsequent periods of clinical TB were treated as the same infection period.
